# Evaluation of Prognostic and Predictive Significance of Circulating MicroRNAs in Ovarian Cancer Patients

**DOI:** 10.1155/2017/3098542

**Published:** 2017-02-15

**Authors:** Ann Rita Halvorsen, Gunnar Kristensen, Andy Embleton, Cybil Adusei, Maria Pilar Barretina-Ginesta, Philip Beale, Åslaug Helland

**Affiliations:** ^1^Department of Cancer Genetics, Institute for Cancer Research, Oslo University Hospital, Radiumhospitalet, Oslo, Norway; ^2^Department of Gynecologic Oncology, Institute for Cancer Genetics and Informatics and University of Oslo, Oslo, Norway; ^3^MRC Clinical Trials Unit at UCL, Institute of Clinical Trials & Methodology, London, UK; ^4^DOCs International, Buckinghamshire, UK; ^5^Department of Medical Oncology, Catalan Institute of Oncology, Doctor Josep Trueta University Hospital, Girona, Spain; ^6^Department of Medical Sciences, Medical School, University of Girona, Girona, Spain; ^7^Medical Oncology, Royal Prince Alfred Hospital and Concord Repatriation General Hospital and Chris O'Brien Lifehouse, Sydney, NSW, Australia; ^8^Department of Oncology, Oslo University Hospital, Radiumhospitalet, Oslo, Norway

## Abstract

Ovarian cancer patients are recognized with poor prognosis. This study aimed to identify microRNAs in plasma for predicting response to treatment and outcome. We have investigated microRNAs in plasma from ovarian cancer patients enrolled in a large multicenter study (ICON7), investigating the effect of adding bevacizumab to standard chemotherapy in patients diagnosed with epithelial ovarian cancer. Patients with different histology, grade, and FIGO stages were included (*n* = 207) in this study. Screening of 754 unique microRNAs was performed in the discovery phase (*n* = 91) using TaqMan Low Density Arrays. The results were validated using single assays and RT-qPCR. Low levels of miR-200b, miR-1274A (tRNA^Lys5^), and miR-141 were significantly associated with better survival, confirmed with log-rank test in the validation set. The level of miR-1274A (tRNA^Lys5^) correlated with outcome was especially pronounced in the high-grade serous tumors. Interestingly, low level of miR-200c was associated with 5-month prolongation of PFS when treated with bevacizumab compared to standard chemotherapy. We found prognostic significance of miR-200b, miR-141, and miR-1274A (tRNA^Lys5^) in all histological types, where miR-1274A (tRNA^Lys5^) may be a specific marker in high-grade serous tumors. The level of miR-200c may be predictive of effect of treatment with bevacizumab. However, this needs further validation.

## 1. Introduction

Chemoresistance is an obstacle for effective treatment of ovarian cancer. The majority of the ovarian cancer patients are diagnosed at late stage with poor overall survival [1], due to lack of symptoms in early stage. For patients with spread of disease, the survival rate is low with standard chemotherapy [2]. Despite development of new drugs, many patients do not benefit from the treatment with these drugs. As of today, patients responding to given treatment cannot be distinguished from those without response prior to administration.

Epithelial ovarian cancer can be divided into 5 histological subtypes: high-grade serous, low-grade serous, mucinous, clear cell, and endometrioid, of which high-grade serous carcinomas are the most common and most studied histological type [2].

During tumor development, the tumor vascularization is essential in order to secure a persistent supply of blood, oxygen, and nutrition to sustain the rapid rate of growth of tumor cells. The tumor angiogenesis is controlled by a large number of proangiogenic factors such as transforming growth factor beta 1 (TGF-*β*1), transforming growth factor alpha (TGF-*α*), and vascular endothelial growth factor (VEGF). The latter has been identified as the key mediator and the VEGF family consists of six members: VEGF-A, placenta growth factor (PIGF), VEGF-B, VEGF-C, VEGF-D, and VEGF-E [3]. It is suggested that, by inhibiting VEGF, the vascular permeability can be reduced leading to a reduction in the tumor interstitial pressure, hence improving the delivery of chemotherapeutic drugs to the tumor [4].

Bevacizumab (anti-VEGF) is a recombinant humanized monoclonal antibody which binds to all the isoforms of VEGF-A [5]. Bevacizumab is approved by health authorities (EMA and FDA) to be used in combination with chemotherapy in a number of cancer types [6]. In ovarian cancer, bevacizumab is approved in combination with chemotherapy and as monotherapy maintenance after chemotherapy. In a phase 3 randomized trial on ovarian cancer patients (ICON7), high-risk patients showed prolonged progression free survival (PFS) and overall survival (OS) when bevacizumab was given together with standard chemotherapy followed by a maintenance phase compared with standard chemotherapy alone. This effect on survival was not seen among the non-high-risk patients [7]. This underlines the importance of discriminating between responders and nonresponders to improve the outcome. In order to pinpoint patients benefitting from the treatment, blood based predictive biomarkers might be essential supplement for the clinicians when type of treatment is chosen [8].

MicroRNAs are small nucleotides established as regulators of mRNA translation and hence involved in both normal and pathological processes in a cell. Dysregulation of microRNAs has been reported in all stages and types of cancer, and it has been demonstrated that microRNAs are involved in mechanisms such as tumor growth, invasion, angiogenesis, and immune evasion [9]. Recently, microRNAs have been detected in biofluids such as blood, urine, saliva, and breast milk [10]. In biofluids, these molecules are exceptionally stable due to protection by proteins and extracellular vesicles [11, 12]. This makes these molecules suitable as biomarkers for cancer diagnosis, prognosis, and prediction of effect of therapy. Circulating microRNAs as biomarkers have several advantages compared with tissue-based markers. Blood is easy to obtain; microRNA analyses are fast and cheap and can be done repeatedly to monitor the disease. Previous studies on tumor tissue have shown that microRNAs are involved in chemoresistance [13]. In a study of paclitaxel resistance in cultured ovarian cancer cells, overexpression of 17 microRNAs was detected in the resistant cells, and two of these were also significantly associated with prognosis. Further, downregulation of 4 microRNAs was found in the paclitaxel sensitive cells [14].

The purpose of this study was to identify circulating microRNAs able to identify ovarian cancer patients in high risk for relapse. Further, the goal was to identify candidate circulating microRNAs predictive of benefit from treatment with bevacizumab so that toxicity and costs can be reduced for patients with low chance of response.

## 2. Material and Methods

Ovarian cancer patients were enrolled in the ICON7 study, a randomized, 2-arm, multicenter study designed to evaluate the safety and efficacy of adding bevacizumab to standard chemotherapy with carboplatin and paclitaxel, as previously described [7].

Alongside the clinical study, patients were asked to participate in a translational study. Research projects, utilizing ICON7 translational research samples, underwent peer review and were approved by the Trial Management and Steering Committees and the Ethics Committee in charge of the trial.

Baseline blood samples (*n* = 207) were collected in EDTA monovettes prior to administration of chemotherapy, centrifuged (2000*g* for 10 minutes at 20°C) within 30 minutes of venepuncture, aliquoted, and stored at −80°C according to standard operating procedures. The plasma samples were shipped to the central sample bank at the University of Leeds, UK (R Banks), where they were stored anonymously at −80°C.

In order to identify microRNAs associated with progression free survival, samples in the upper and lower quartile of progression free survival range were selected (*n* = 91) for the discovery cohort (Table 1). The validation cohort consisted of the remaining patients (*n* = 116). To test the predictive potential of the microRNAs, the samples were also stratified according to given treatment: standard chemotherapy and standard chemotherapy plus bevacizumab were called chemo group and bevacizumab group, respectively. In this study, grade 2 and 3 serous carcinomas are defined as high-grade.

### 2.1. MicroRNA Analysis

RNA was extracted from 500 *μ*L plasma using miRCURY™ RNA isolation kit for biofluids (cat# 300113, Exiqon, Denmark) as prescribed in the protocol. Then, 30 ng totRNA was reverse-transcribed using megaplex™ RT primer pool (pools A and B, cat# 4444745, Agilent Technologies, Santa Clara, CA) and TaqMan® MicroRNA reverse transcription kit (cat# 4366596, Agilent Technologies, Santa Clara, CA). In order to increase the sensitivity, preamplification of the cDNA was implemented using megaplex preamp primer pool (pools A and B, cat# 4444748, Life Technologies) and TaqMan preamp master mix (cat# 4391128, Agilent Technologies, Santa Clara, CA). The samples were then mixed with TaqMan Universal PCR Master Mix, NoAmpErase® UNG, 2X, and applied to the TaqMan Low Density Arrays (TLDA, cat# 4444913, Agilent Technologies, Santa Clara, CA). The analysis was performed at 7900HT Fast Thermocycler System (Agilent Technologies, Santa Clara, CA). The protocol from supplier (PN 4399721) was followed for all the procedures. The raw C_T_ data were exported to the ExpressionSuite software (v.1.0.3, Applied Biosystem, Foster City, CA) for global normalization and quality control. For the validation, the microRNAs of interest were first reverse-transcribed and then quantified using single assays and RT-qPCR. The procedures were performed according to standard protocols. The procedures were performed according to standard protocols. The primers, reaction mix components, and temperature conditions used for reverse transcription and qPCR are listed in Supplementary Table 1 in Supplementary Material available online at https://doi.org/10.1155/2017/3098542. All samples were run in triplicate. The comparative threshold (Ct) was used to evaluate the relative detection level of each microRNA. Both negative and positive controls were included. Pooled plasma from women diagnosed with ovarian cancer was used as positive control.

### 2.2. Normalization

The raw C_T_ data were exported to the ExpressionSuite software (v.1.0.3, Life Technologies) for normalization and quality control. Threshold and baseline were automatically calculated for each assay, and a global normalization was performed as recommended for large scale microRNA expression profiling [15]. The expression of each microRNA was then mean centered using only expressed microRNAs [16]. Normalized ΔC_T_ data were used for calculation of relative gene expression in fold change (2^−ΔΔCt^). For validation of the selected microRNAs, the four most uniformly expressed microRNAs (miR-220, miR-19b, sU6, and miR-320) from the TLDA cards were chosen as reference genes for normalization. To obtain ΔC_T_, the mean expression level of the four microRNAs (C_T  ref_) was subtracted from the mean Ct level (C_T  target_) of each sample. Due to low expression of microRNAs in plasma, missing values were most likely the results of too few RNA-copies. No detected measurements were replaced with zero in linear scale. For the logarithmic scale, the lowest expressed value for each microRNA replaced the missing values [17]. MicroRNAs detected in less than 30% of the samples were removed. Preamplification was not utilized in the validation and Ct values of <40 indicated the presence of microRNA.

### 2.3. Statistics

Significance analysis of microarrays (SAM) was performed in J-express to identify microRNAs with differential abundance in groups of interest [18]. Kaplan-Meier survival analysis, log-rank test, and multivariate Cox regression analysis were performed in SPSS (IBM SPSS Statistics, Version 21.0. Armonk, NY: IBM Corp). In the Cox multivariate analysis histology, grade, FIGO stage, residual disease, type of treatment, and age were included. PFS was calculated from the date of randomization to the date of disease progression or death, whichever occurred first. Patients who were still alive without progression were censored at the date of their last assessment. Student's *t*-test was applied when applicable. Statistical significance was considered with *p* < 0.05 or for multiple testing, false discovery rate (FDR) < 0.05.

## 3. Results

### 3.1. Study Design and Patient Characteristics

Totally, we used plasma samples collected from 207 patients with epithelial ovarian cancer. For patients selected for the discovery set, the group with long PFS had a median survival of 29 months and the group with short PFS had a median PFS of 7.8 months. The median PFS for patients in the validation set was 17 months. The patient characteristics for the two sets are given in Table 1. Treatment with bevacizumab had no prognostic impact in the discovery set but was of independent prognostic significance for PFS in the validation set (*p* < 0.001, HR = 0.37, CI 95% = 0.23–0.60).

### 3.2. Selection of miRNA Associated with Prognosis

In the discovery set, 754 unique microRNAs were profiled. We were able to measure 403 different microRNAs in more than 30% of the samples, whereas 104 microRNAs were detectable in all the samples.

**Figure 1:**
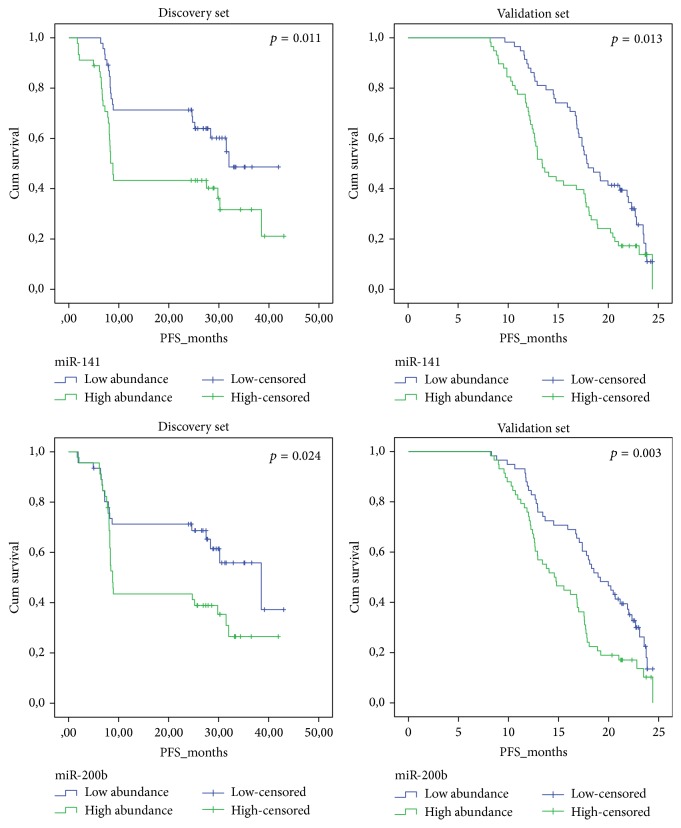
The prognostic potential of miR-200b and miR-141 is displayed using Kaplan-Meier survival plot and log-rank test was performed on data obtained from the discovery set and the validation set. High abundance means abundance above median and low abundance means under median. PFS = progression free survival in months. Cum survival = cumulative survival.

In the discovery set, we found 6 microRNAs (miR-1274A, miR-141, miR-200b, miR-200c, miR-520c-5p, and miR-520d-5p) with significantly higher abundance in patients reported with short PFS compared with those with long PFS (SAM analysis, FDR < 0.001, Table 2). The log-rank test revealed 4 microRNAs (miR-1274A, miR-141, miR-200b, and miR-200c) significantly associated with survival. In the validation set, the log-rank test confirmed the prognostic association for miR-141 and miR-200b (Table 2 and Figure 1). In the multivariate Cox regression test, miR-200b obtained significance (*p* = 0.006, HR = 0.79, CI 95% = 0.68–0.94) and miR-1274A was of borderline significance (*p* = 0.085, HR = 0.85, CI 95% = 0.70–1.02), while miR-141 did not obtain significance (*p* = 0.153, HR = 0.91, CI 95% = 0.81–1.03). Type of treatment, FIGO stage, and residual disease all obtained significance (Table 3).

Since high-grade serous carcinoma is the largest group, the same statistical analyses were applied to this group. In the high-grade serous carcinomas, low levels of miR-1274A were significantly associated with prolonged survival in the log-rank test both in the discovery set (*p* = 0.031) and in the validation set (*p* = 0.012). The same tendency was seen for miR-141 and miR-200c, although this was not significant in the validation set (Table 2). In the multivariate Cox regression analysis on the validation set, miR-1274A obtained independent significance (*p* = 0.041, HR = 0.78, CI 95% = 0.62–0.99), while miR-141 and miR-200b did not obtain independent significance (*p* = 0.69 and *p* = 0.15, resp.). The remaining histological groups were too small for group specific analyses. The level of miR-520d-3p and miR-520c-3p was also analysed in the validation set, but, due to very low abundance and many cases with no detected signals, these microRNAs were not included in further analyses.

### 3.3. Evaluation of MicroRNA as a Possible Predictive Factor

**Figure 2:**
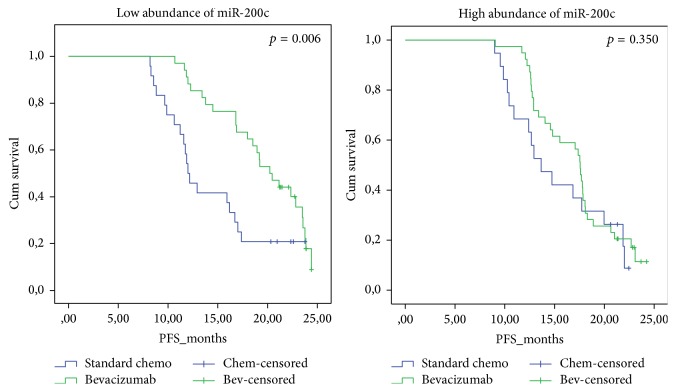
The Kaplan-Meier plot shows that low levels of miR-200c are associated with significantly better survival when bevacizumab is added to standard chemotherapy, compared to standard chemotherapy alone. Standard chemotherapy is carboplatin and paclitaxel. PFS = progression free survival in months. Cum survival = cumulative survival. High levels of miR-200c were not predictive of therapy.

To evaluate the predictive potential of the microRNAs, we checked if there was significantly different time to progression between the two treatment groups related to the level of the microRNAs in plasma. We stratified the microRNAs based on high and low abundance with the median as cutoff. In the discovery group, we did not see any difference in PFS between the two treatments groups related to abundance of the microRNAs. In the validation set, miR-200c were associated with significantly better survival in the log-rank test when treated with bevacizumab, compared to standard chemotherapy with a median PFS of 19.5 months and 14.5 months, respectively (*p* = 0.006, Figure 2). This was confirmed by multivariate Cox regression (*p* < 0.0001, HR = 4.33, CI 95% = 1.96–9.58). No significant differences between treatments were seen with high levels of miR-200c.

Low levels of miR-200b and miR-141 were associated with significantly better PFS in the log-rank test when treated with bevacizumab compared to standard chemotherapy, but this was not confirmed when adjusting for explanatory variables in multivariate Cox regression analysis.

## 4. Discussion

We identified 4 microRNAs (miR-200b, mir-200c, miR-1274A, and miR-141) significantly associated with survival in the discovery set. The prognostic significance of miR-200b and miR-141 was confirmed in the validation set using the log-rank test. When adjusting for other explanatory variables, level of miR-200b was significant, and miR-1274A and miR-141 were of borderline significance. When evaluating the high-grade serous group alone, miR-1274A was found of independent prognostic significance. In these evaluations, the small number of patients should be taken into account.

We believe that biomarkers measured in blood are of great value since it is less invasive, easy to obtain, cost-effective, and fast. On the other hand, there are also challenges in working with microRNAs in blood. The level of circulating microRNAs can be very low, and blood contains several components which may influence the analysis. Therefore, when working with microRNAs, choice of method is crucial. In this study, quantitative real-time PCR, known as a very robust, accurate, and sensitive method, was chosen [19]. To date, several microRNA candidates have been identified as diagnostic, prognostic, or predictive biomarkers [20] in ovarian cancer patients. However, most of these studies have been performed in ovarian tissue or cell lines and with contradictory results. Interestingly, Chen et al. investigated 8 published microRNA expression studies in ovarian cancer and revealed that four differentially expressed microRNAs (miR-200a, miR-141, miR-200b, and miR-200c), all members of the miR-200 family, were consistently upregulated in tumor samples in four or more of the studies [21]. Moreover, the level of miR-141, miR-200b, and miR-200c derived from exosomes in blood has been reported to correlate with the level in ovarian tumor cells [22]. This is in line with our results where the level of miR-141 and miR-200b was elevated in ovarian cancer patients with poor survival compared to those with longer PFS in both the discovery and the validation set. The prognostic potential of miR-200 family has been elucidated and validated in numerous of cancer diseases including ovarian cancer, colorectal cancer, and lung cancer [23–25].

In a recent study, miR-1274A was found elevated in plasma from patients diagnosed with serous epithelial ovarian cancer [26]. A large number of microRNAs have recently been refined in the miRBase and only 1/3 of the first annotated microRNAs seem to be real microRNAs [27]. Recent data suggests that miR-1274A probably is a fragment from the 3′ end of Lys tRNA (tRNA^Lys5^) [28]. Interestingly, tRNA fragments have proven to have many different function, including a regulatory capacity [29]. Moreover, miR-1274 (tRNA^Lys5^) is found with high amounts in extracellular vesicles derived from human breast cancer cells [30].

When this study was initiated, plasma from individuals with very different time to progression was selected for the discovery set and the remaining samples were later supplied for validation. The remaining samples came from individuals with minor differences in time to progression. Another difference between the two data sets was the use of preamplification. This was implemented in order to increase the sensitivity of microRNAs in the discovery phase but was not done in the validation set. In the discovery set, two of the identified microRNAs (miR-520c-3p and 520d-3p) were detected at very low levels in only 38% and 67% of the samples, respectively. These were excluded for further analysis.

In the discovery set, miR-200c was the microRNA most significantly associated with survival. However, this was not confirmed in the validation set. This might indicate that the prognostic potential of this microRNA is detected only in samples with high variance in survival. The possible prognostic role of miR-200c should be further explored in an independent study. In a study of microRNAs in serum of epithelial ovarian cancer, miR-200b and miR-200c showed high levels in serum compared with normal controls. In addition, the level of miR-200c was associated with progression of the disease [23]. Despite two very different data sets, we did identify microRNAs with prognostic potential. Bearing the differences in the two data sets and the small numbers in mind, the prognostic value of miR-1274A seems robust, at least in high-grade serous ovarian carcinoma, and a prognostic value of miR-141and miR-200b seems likely.

To further test if any of the microRNAs were predictive of therapy response, we assumed that either low or high levels of the microRNAs would show different time to progression between the treatment groups. In the discovery set, no differences were seen. However, in the validation set, low level of miR-200c was significantly associated with better survival when treated with bevacizumab compared to standard chemotherapy. This was still significant after correcting for explanatory variables. Treatment with bevacizumab had no prognostic impact in the discovery set, which may explain that no microRNA had predictive value in this set. Our findings may indicate that low level of miR-200c in plasma is a predictive marker for pinpointing the patients benefitting from adding bevacizumab to the treatment. However, validation of the predictive potential of miR-200c is necessary. Interestingly, members of the miR-200 family have been identified as pivotal in several malignant processes, such as epithelial to mesenchymal transition (EMT), angiogenesis, and apoptosis [31–33]. In a study of colorectal cell lines, miR-200c was involved in regulation of migration and tube formation during angiogenesis [32]. This may explain the relation between miR-200c in plasma and response to the antiangiogenesis drug, bevacizumab, we found in our study.

Studies of microRNAs predictive of treatment have mostly been performed in ovarian tissue, such as low levels of miR-378 reported to be associated with better response to bevacizumab [34] and a signature of 3 microRNAs predicting chemoresistance in serous epithelial ovarian carcinomas [13]. Recently, in matched biopsies from ovarian cancer patients, four microRNAs were associated with prognosis and response to platinum-based neoadjuvant chemotherapy response [35]. Few studies have so far identified circulating microRNAs in this context. However, in a study of colorectal cancer, circulating levels of miR-126 have been found to predict treatment response to chemotherapy and bevacizumab [36].

A shift in treatment strategies for ovarian cancer patients is evolving, from treating the majority of patients similarly to a more personalized treatment based on molecular aberrations [37]. The response to a given treatment can be difficult to predict upfront and the most efficient therapy might not be given. Despite accumulating data of the biology involved in chemosensitivity and chemoresistance, markers for pinpointing the patients who will benefit from a given therapy are lacking [38]. Robust prognostic markers in blood would help clinicians to identify patients at higher risk of relapse and tailor the treatment regimen or spare the patients at low risk the side effects of treatment [39].

## 5. Conclusion

We identified miR-200b as a robust prognostic marker able to detect ovarian cancer patients with high risk of relapse independent of stage, histology, and residual tumor after surgery. We found miR-141 to be a potential prognostic marker. In addition, miR-1274A was a prognostic marker in patients with high-grade serous ovarian cancer. We will also propose miR-200c as a potential circulating biomarker for prediction of better outcome after treatment with bevacizumab in combination with standard chemotherapy. These results need further validation for clinical utility. This is the first study to identify plasma microRNAs potentially able to predict response to bevacizumab.

## Supplementary Material

Supplementary Table 1: A: RT Reaction mix components. B: Real Time PCR reaction mix components. C: Reverse Transcription Reaction (30 ng totRNA input). D: Primer sequences for different miRNAs.

## Figures and Tables

**Table 1:** Clinical information of all patients included in this study of microRNAs is given in the table. In the discovery set, samples collected from patients with long PFS and short PFS are selected from the upper and lower quartile of PFS, respectively. Samples in the validation set are recognized with less variation in survival time.

	Discovery set				Validation set	
	Bevacizumab long PFS	Standard chemotherapy long PFS	Bevacizumab short PFS	Standard chemotherapy short PFS	Bevacizumab	Standard chemotherapy
Number of samples	*n* = 25	*n* = 26	*n* = 16	*n* = 24	*n* = 73	*n* = 43
Age (average)	56.4	57.1	56.6	59.8	53.8	58.1
Histology						
*Serous*	16	17	11	14	49	30
*Mucinous*	0	0	0	2	0	0
*Endometrioid*	1	3	0	2	2	4
*Clear cell*	3	4	4	2	10	4
*Mixed*	4	2	1	1	10	3
*Other*	1	0	0	3	2	2
FIGO						
*IA/IB/IC*	4	5	0	0	5	3
*IIA/IIB/IIC*	7	7	2	0	6	4
*IIIA/IIIB/IIIC*	13	14	10	18	51	32
*IV*	1	0	4	6	11	4
Grade						
* 1*	2	1	0	0	5	2
* 2*	2	4	1	5	11	6
* 3*	19	21	15	17	57	34
* Unknown*	2	0	0	2		1
PFS median (months)	28.8	29.4	8.1	7.2	18	12.9
Residual disease						
* Inoperable*	0	0	1	3	1	2
* Residual > 1 cm*	3	4	6	12	24	17
* Residual ≤ 1 cm*	5	2	6	5	20	6
* Residual 0 cm*	17	20	3	3	28	18

**Table 2:** The levels of 6 identified microRNAs were investigated for association with survival irrespective of treatment group, in the discovery set and in the validation set. False discovery rate (FDR) is shown in the discovery set, and the *p* values from Student's *t*-test are applied in the validation set. The prognostic value of the six microRNAs was also tested in all the histology types combined, in addition to high-grade serous samples separately. NA = not applicable and NS = not significant.

	Discovery set			Validation set		
Histology	All histologies combined (*n* = 91)		High-grade serous (*n* = 58)	All histologies combined (*n* = 116)		High-grade serous (*n* = 71)
MicroRNA	SAM analysis	Log-rank test	Log-rank test	Student's *t*-test	Log-rank test	Log-rank test
	FDR	*p* value	*p* value	*p* value	*p* value	*p* value
miR-1274A	<0.001	0.002	0.031	0.61	0.48	0.012
miR-141	<0.001	0.011	0.111	0.012	0.013	0.099
miR-200b	<0.001	0.024	0.01	0.014	0.003	0.288
miR-200c	<0.001	0.001	0.007	0.092	0.305	0.17
miR-520d-3p	<0.001	NS	NA	0.343	NA	NA
miR-520c-3p	<0.001	NS	NA	0.742	NA	NA

**Table 3:** Cox regression analysis shows the variables significantly associated with prognosis. Variables not significant are left out of the final model except from miR-1274A and miR-141. In the full model, the variables including age, FIGO stage, grade, residual disease, and type of treatment were included in addition to the 4 microRNAs.

Variable	*p* value	HR	95% CI for HR
mir_1274B	0.085	0.846	0.70–1.02
miR_200b	0.006	0.798	0.68–0.94
miR-141	0.153	0.914	0.81–1.03
FIGO stage			
Stage I (reference)	0.08		
Stage II	0.911	1.073	0.31–3.68
Stage III	0.352	1.564	0.61–4.01
stage IV	0.036	3.368	1.08–10.49
Residual disease			
No residual disease (reference)	<0.001		
Residual 0-1 cm	0.853	0.863	0.18–4.13
Residual > 1 cm	<0.001	3.005	1.70–5.31
Inoperable	<0.001	3.259	1.76–6.04
Treated with bevacizumab versus chemotherapy	<0.001	0.373	0.23–0.60
