# Supplementary material for: Evaluation of Prognostic and Predictive Significance of Circulating MicroRNAs in Ovarian Cancer Patients
Source: Dis Markers. 2017 Feb 15;2017:3098542. doi: 10.1155/2017/3098542 (PMC5331307; doi:10.1155/2017/3098542)
Supplement: Supplementary file 1 — Supplementary Table 1: A: RT Reaction mix components. B: Real Time PCR reaction mix components. C: Reverse Transcription Reaction (30 ng totRNA input). D: Primer sequences for different miRNAs. [file 3098542.f1.pdf]

Supplementary Tables

Supplementary Table 1

A: RT Reaction mix components.

Component Master mix volume 10 µl reaction	1x µl
100mM dNTPs (with dTTP)	0.10
MultiScribe™Reverse Transcriptase, 50 U7µL	0.67
10x Reverse Transcription Buffer	1.00
Rnase Inhibitor, 20 U/µL	0.13
Nuclease-free water	2.80
Total volume	4.70
3µl RT primer (spesific for each miRNA)(5X)	2.00
3.3µl sample (30ng)	3.30

B: Real Time PCR reaction mix components.

Master mix volume 10µl	1x
TaqMan®Small RNA Assay (20x)	0.50
Product from RT reaction	0.70
TaqMan®Universal PCR Master Mix(2x),noUNG	5.00
Nuclease-free water	3.80
Total volume	10.00

C. Reverse Transcription Reaction (30ng totRNA input)

Step type	Times (minutes)	Temp
Hold	30	16°C
Hold	30	42°C
Hold	5	85°C
Hold	∞	4°C

D. Primer sequences for different miRNAs

miRNA	assay ID	sequence
miR-141	463	UAACACUGUCUGGUAAAGAUGG
miR-200c	2300	UAAUACUGCCGGGUAAUGAUGGA
miR-200b	2251	UAAUACUGCCUGGUAAUGAUGA
miR-1274A	2883	GUCCCUGUUCAGGCGCCA
miR-520d-3p	2743	AAAGUGCUUCUCUUUGGUGGGU
miR-520c-3p	2400	AAAGUGCUUCCUUUUAGAGGGU
